# Effects of Calcium and Magnesium Fertilization on the Rhizosphere Bacterial Community Assembly and Specific Biomarkers in Rainfed Maize

**DOI:** 10.3390/plants15010060

**Published:** 2025-12-24

**Authors:** Zhaoquan He, Xue Shang, Xiaoze Jin

**Affiliations:** 1 School of Life Sciences, Yan’an University, Yan’an 716000, China; 2 Shaanxi Key Laboratory of Research and Utilization of Resource Plants on the Loess Plateau, College of Life Sciences, Yan’an University, Yan’an 716000, China; 3 Key Laboratory of Applied Ecology of Universities in Shaanxi Province on the Loess Plateau, Yan’an University, Yan’an 716000, China; 4 Northwest Institute of Eco-Environment and Resources, Chinese Academy of Sciences, Lanzhou 730000, China

**Keywords:** calcium and magnesium addition, rhizosphere bacterial community, dryland farming, microbial biomarkers, community assembly

## Abstract

This study investigated the effects of varying levels and combinations of calcium (Ca) and magnesium (Mg) supplementation on the diversity, composition, and species differentiation of the rhizosphere soil bacterial community in rainfed maize, aiming to reveal their regulatory mechanisms on the rhizosphere micro-ecosystem. A field micro-plot experiment was conducted with seven treatments: low Ca (U), high Ca (V), low Mg (W), high Mg (X), low Ca and low Mg (Y), high Ca and high Mg (Z), and a control (K, no supplementation). The bacterial communities were analyzed using high-throughput sequencing of the 16S rRNA gene, and the data were processed using the QIIME2 pipeline, as well as multivariate statistical analyses, and LEfSe. The main results demonstrated that Ca and Mg supplementation significantly altered the rhizosphere bacterial community structure (beta-diversity). Analysis of Similarities (ANOSIM) indicated significant differences between treatments (R > 0.4, *p* < 0.01). LEfSe analysis successfully identified key biomarkers responsive to different treatments. For instance, high Ca treatment significantly enriched the genus Clostridium within the phylum Firmicutes, whereas high Mg treatment specifically enriched the genus *Lysobacter*. Furthermore, Ca-Mg interactions exhibited non-additive effects, and the coupled Ca-Mg supplementation treatments (Y, Z) formed unique species assemblages. As key environmental drivers, Ca and Mg supplementation specifically reshaped the rhizosphere bacterial community through “environmental filtering” in rainfed maize. This study provides a theoretical basis for understanding the microbiological pathways through which secondary element fertilizers influence soil health, offering practical implications for precisely managing rhizosphere micro-ecology through Ca and Mg supplementation to promote the sustainable development of dryland farming.

## 1. Introduction

Dryland agriculture is a critical component of global food production, whose sustainability is heavily reliant on soil health and fertility. Among essential soil nutrients, calcium (Ca) and magnesium (Mg), as secondary macronutrients, play dual roles: they are directly involved in plant physiological metabolism, maintaining cell wall and membrane stability, and act as key regulators of soil physicochemical properties such as pH, aggregate structure, and cation exchange capacity [1,2]. In recent years, with the integration of chemical ecology and microbial ecology, the rhizosphere—a microzone of soil strongly influenced by root activities—has become a hotspot for researching nutrient cycling and microbial interactions. The rhizosphere, a narrow zone of soil influenced by root exudates and activities, is a dynamic hotspot for microbial life and nutrient cycling. Rhizosphere microorganisms, particularly bacteria, function as the “engine” of the soil ecosystem, driving vital processes including organic matter decomposition, nutrient mineralization and solubilization (e.g., of phosphorus and micronutrients), phytohormone production, and pathogen suppression [3,4]. The structure and function of these bacterial communities are pivotal determinants of soil fertility and plant health. Therefore, investigating how Ca and Mg supplementation alters the rhizosphere microenvironment and subsequently influences the assembly and succession of bacterial communities is not only of theoretical importance for understanding the response mechanisms of soil microbes, but also provides a scientific basis for optimizing nutrient management strategies in dryland agriculture and achieving the green agricultural goal of enhancing soil fertility through microecological regulation [5,6].

Nutrient management, including the application of Ca and Mg, can significantly alter the rhizosphere microenvironment. These changes act as environmental filters, shaping microbial community assembly [7]. For instance, several studies have reported that Ca supplementation generally increases the relative abundance of Actinobacteria and Firmicutes while potentially reducing the abundance of *Acidobacteria*, largely through raising soil pH [8]. Practically, these shifts in microbial communities have been linked to potential improvements in soil carbon sequestration efficiency, nitrogen use efficiency, and suppression of soil-borne diseases [9,10]. Furthermore, studies on maize rhizosphere microbiota have indicated that root exudates are a core driver shaping rhizosphere communities, and nutrient availability can indirectly exert selective pressure on microbes by altering exudate composition. Collectively, these findings form the theoretical foundation for understanding the “soil-nutrient-microbe-plant” interaction network, confirming that nutrient management is an effective lever for regulating soil microecology [11,12].

Despite these advances, a deeper analysis of the field reveals both contrasting viewpoints and significant limitations [13]. Firstly, there are notable disparities in the reported effects of Ca and Mg. Most studies have examined the individual effects of either Ca or Mg in isolation, and the conclusions are not always consistent [14]. For example, while some studies emphasize the predominant role of Ca-mediated pH adjustment, others have found that Ca^2+^ and Mg^2+^ ions can directly influence microbial physiology and competitive behaviors as signaling molecules or intracellular messengers, even when pH changes are minimal [15,16]. This inconsistency highlights the complexity of the underlying mechanisms, suggesting the possible existence of both pH-dependent and pH-independent pathways [17]. Secondly, a prevalent trend and core limitation is that existing research predominantly focuses on single-element effects or high-level fertilization impacts on bulk soil microorganisms, overlooking two critical aspects: firstly, the potential synergistic or antagonistic effects of Ca-Mg interactions on microbes; and secondly, insufficient attention to the rhizosphere, a unique and plant-intimate microzone [18].

These limitations profoundly affect the validity and reliability of research conclusions. Neglecting interaction effects may lead to deviations in predicting the effectiveness of practical field fertilization, which typically involves compound fertilizers [19]. Moreover, conflating the rhizosphere with bulk soil prevents the precise capture of responses from the microbial fraction most critical to plant nutrition and health. Consequently, conclusions derived from bulk soil studies may not be directly extrapolated to the rhizosphere micro-ecosystem, thereby limiting their potential for guiding precision agriculture [20,21].

In summary, clear knowledge gaps remain in dryland agricultural systems: how do different levels and combinations of Ca and Mg supplementation specifically affect the diversity, composition, and structure of the bacterial community in maize rhizosphere soil, and drive the enrichment of key differential species (biomarkers)? Therefore, we hypothesized that the application of Ca and Mg fertilizers would significantly alter the rhizosphere bacterial community structure and diversity in rainfed maize by modifying the soil microenvironment, and that these changes would be non-additive when both elements are applied together. This study aims to systematically investigate the effects of different levels and combinations of Ca and Mg supplementation on the rhizosphere bacterial community in rainfed maize. The specific objectives are as follows: (1) To determine how Ca and Mg supplementation alters the alpha (within-sample) and beta (between-sample) diversity of the rhizosphere bacterial community. (2) To identify successional changes in the composition and structure of the bacterial community under different Ca and Mg treatments. (3) To identify key bacterial taxa (biomarkers) that respond specifically to different Ca and Mg treatments and to infer their potential ecological functions, including possible roles in micronutrient dynamics.

Elucidating these issues will deepen our understanding of Ca and Mg fertilizer effects from a microbial ecology perspective, providing a novel theoretical basis for developing precise nutrient management strategies in dryland agriculture that enhance both crop productivity and soil ecosystem health.

## 2. Materials and Methods

### 2.1. Experimental Design

#### 2.1.1. Study Site

The experiment was conducted at the Ansai District Ecological Agriculture Technology Innovation Experimental Demonstration Station, a field scientific observation and research station of the College of Life Sciences, Yan’an University. The station is located in Ansai District, Yan’an City, Shaanxi Province, China (36°51′30″ N, 109°19′23″ E). This region is characterized by a typical dryland farming system with no irrigation facilities. The mean annual precipitation is 540 mm, primarily concentrated from July to September [22]. The mean annual air temperature is 8.8 °C, with an annual sunshine duration of 2416 h per year and a frost-free period of 143–174 days. The experimental area features widely exposed loess parent material and is dominated by homogeneous *Calcaric Regosols*, with a sandy loam texture. The fundamental soil physicochemical properties of the plow layer (0–20 cm depth), determined before the experiment establishment, were as follows: pH 8.5 (measured potentiometrically in a 1:2.5 soil: water suspension), soil organic matter 6.36 g∙kg^−1^ (by the K_2_Cr_2_O_7_ oxidation method), total nitrogen 0.83 g∙kg^−1^ (by the Kjeldahl method), total phosphorus 0.57 g∙kg^−1^ (by HClO_4_–H_2_SO_4_ digestion and molybdenum blue colorimetry), total potassium 18.20 g∙kg^−1^ (by HF–HClO_4_ digestion and flame photometry), available phosphorus 15.30 mg∙kg^−1^ (extracted with 0.5 M NaHCO_3_, pH 8.5), available potassium 141.35 mg∙kg^−1^ (extracted with 1 M NH_4_OAc, pH 7.0), exchangeable calcium 4.8 cmol_(+)_∙kg^−1^, and exchangeable magnesium 1.2 cmol_(+)_∙kg^−1^ (both extracted with 1 M NH_4_OAc, pH 7.0, and determined by ICP-OES), indicating uniform soil fertility with a baseline level of Ca and Mg [23].

#### 2.1.2. Experimental Design Scheme

A single-factor randomized block design was employed. The application rates for calcium (Ca) and magnesium (Mg) were determined based on: (i) the critical deficiency levels and common sufficiency ranges for maize reported in regional agronomic guides for dryland farming on the Loess Plateau; (ii) the baseline exchangeable Ca and Mg content of our experimental soil (Table 1), aiming to create a clear gradient from deficiency correction to surplus addition; and (iii) practical fertilization ranges reported in previous studies investigating secondary macronutrient effects in similar cropping systems [24]. Accordingly, three calcium (Ca) levels (None: 0.00 kg ha^−1^, Low: 24.50 kg ha^−1^, High: 49.00 kg ha^−1^) and three magnesium (Mg) levels (None: 0.00 kg ha^−1^, Low: 17.50 kg ha^−1^, High: 35.00 kg ha^−1^) were established, resulting in seven combined Ca and Mg fertilization treatments. Each treatment was replicated four times, resulting in a total of 28 experimental plots (7 treatments × 4 replicates). The specific treatments were: ① Low Ca (U); ② High Ca (V); ③ Low Mg (W); ④ High Mg (X); ⑤ Low Ca & Low Mg (Y); ⑥ High Ca & High Mg (Z); and ⑦ No Ca and No Mg (Control, K). A high-yielding, disease-resistant, highly adaptable, and fast-desiccating spring maize variety, ‘Haodan 168’, was selected as the test crop [25].

Calcium and magnesium were applied as sugar-alcohol chelated calcium (Ca 180 g∙L^−1^) and sugar-alcohol chelated magnesium (Mg 120 g∙L^−1^), respectively. These fertilizers, containing exchangeable and water-soluble forms of the nutrients, are characterized by high absorption efficiency and are environmentally benign. These chelated forms were selected to ensure high nutrient availability and mobility in the high-pH calcareous soil of the experimental site, minimizing rapid precipitation or fixation. The sugar-alcohol chelators (e.g., gluconate or similar) are considered relatively labile and biodegradable organic compounds. While we cannot entirely rule out minor transient effects of the chelating agents on microbial metabolism, their rapid microbial decomposition and the fact that all treatments involving Ca or Mg received an equivalent carrier molecule (with only the central cation differing) allow us to attribute the observed, sustained shifts in microbial community structure primarily to the differential effects of Ca^2+^ and Mg^2+^ ions themselves, rather than to the chelator [26].

Fertilization timing and method: To synchronize nutrient supply with the dynamic demand of maize across its growth stages and to minimize potential leaching or fixation losses in this rainfed system, the total amount of Ca or Mg fertilizer for each treatment was split-applied. The split ratio of 1:2:3:4 across the four key growth stages (seedling-jointing, jointing-tasseling, tasseling-filling, filling-maturity) was designed to provide increasing nutrient inputs aligned with the accelerating growth rate and peak nutrient uptake period during reproductive stages, a strategy adapted from principles of efficient in-season nutrient management for maize. Fertilizers were applied uniformly to the soil around the maize roots on windless, sunny days to ensure sufficient nutrient uptake [27].

#### 2.1.3. Field Management

The experiment comprised 28 plots (7 treatments × 4 replicates), each measuring 6 m × 6 m. All plots utilized ridge-and-furrow cultivation technology, with maize planted in the furrows. The planting density was 60,000 plants ha^−1^. Each plot featured ridges with a width of 20 cm (10 cm for the outermost ridges) and a height of 15 cm, and furrows with a width of 30 cm. Maize plants were spaced 34 cm apart within rows (with the outermost plants 10 cm from the plot edge) and 50 cm between rows [28]. A 4 m buffer zone separated the plots to ensure the accuracy of experimental results. Sowing occurred on 5 May 2024, and harvesting occurred on 28 September 2024, resulting in an average growth period of approximately 150 days. Before the experiment (early-mid April), soil available nitrogen, phosphorus, potassium, and available Ca and Mg contents were measured in the experimental area. Before sowing, a uniform base fertilizer was applied to all plots according to local recommendations for maize: N at 130 kg ha^−1^, P_2_O_5_ at 120 kg ha^−1^, and K_2_O at 38 kg ha^−1^ [29].

#### 2.1.4. Pest, Disease, and Weed Management

Standard agronomic practices were followed for pest, disease, and weed control throughout the maize growth cycle. Weeding was performed manually to avoid chemical interference. No chemical pesticides or biological control agents (e.g., bacterial/fungal inoculants) were applied during the experiment. This protocol was implemented to prevent any confounding effects of pesticidal compounds or introduced microorganisms on the native rhizosphere bacterial community being studied.

### 2.2. Specific Measurements and Methods

#### 2.2.1. Sample Pretreatment

On 28 September 2024, during the maturity stage, plants with relatively good growth and uniform size were selected from each plot. The entire maize plant was carefully uprooted, and soil tightly adhering to the root surface within the 0–5 cm range was collected as rhizosphere soil. Six plants were sampled per treatment, and the rhizosphere soil from these six plants was thoroughly mixed to form one composite sample per plot. Soil samples were immediately placed in sterile sealed bags, stored in a cool box with ice packs (maintaining a temperature of approximately 4 °C), and transported promptly to the laboratory [30]. Samples were transported to the laboratory within 4 h of collection. Upon arrival, the subsample for DNA analysis was transferred to a −80 °C freezer for long-term storage until processing, while the subsample for physicochemical analysis was subjected to air-drying. Each composite sample was divided into two parts: one part was immediately stored at −80 °C for subsequent molecular biological analysis (DNA extraction), and the other part was air-dried at room temperature, ground, and passed through a 2 mm sieve for subsequent analysis of soil bacterial community structure.

#### 2.2.2. DNA Extraction, PCR Amplification, and Sequencing

DNA extraction was performed on the frozen (−80 °C) subsample of the rhizosphere soil. Total genomic DNA was extracted from approximately 0.5 g of soil using the E.Z.N.A.^®^ soil DNA Kit (Omega Bio-tek, Norcross, GA, USA). The purity and concentration of the extracted DNA were assessed by 1% agarose gel electrophoresis and a NanoDrop 2000 spectrophotometer (Thermo Scientific, Wilmington, DE, USA), respectively [31].

The bacterial 16S rRNA gene V3-V4 hypervariable region was amplified by PCR using barcoded specific primers 338F (5′-ACTCCTACGGGAGGCAGCAG-3′) and 806R (5′-GGACTACHVGGGTWTCTAAT-3′), with the extracted DNA as the template. The PCR reaction mixture (20 μL) contained: 4 μL of 5× FastPfu Buffer, 2 μL of 2.5 mM dNTPs, 0.8 μL each of forward and reverse primers (5 μM), 0.4 μL of FastPfu Polymerase, 10 ng of template DNA, and was supplemented with ddH_2_O to a final volume of 20 μL. The amplification program was: 95 °C for 3 min; 27 cycles of 95 °C for 30 s, 55 °C for 30 s, and 72 °C for 45 s; followed by a final extension at 72 °C for 10 min [32].

The PCR products were verified by 2% agarose gel electrophoresis. Target bands were excised and purified using the AxyPrep DNA Gel Extraction Kit (Axygen Biosciences, Union City, CA, USA). After quantification with QuantiFluor™-ST (Promega, Madison, WI, USA), the purified amplicons were sequenced on the Illumina NovaSeq 6000 platform (Majorbio Bio-Pharm Technology Co., Ltd., Shanghai, China) for PE250 sequencing [33].

#### 2.2.3. Bioinformatics Analysis

Raw sequencing reads were quality-filtered using fastp (version 0.19.6) and assembled using FLASH (version 1.2.11) to obtain high-quality clean tags.

Subsequent bioinformatics analysis was performed on the Majorbio Cloud Platform (https://cloud.majorbio.com). The DADA2 algorithm within the QIIME2 (version 2020.2) pipeline was used to process the quality-controlled sequences, performing precise error correction and chimera removal, ultimately yielding Amplicon Sequence Variants (ASVs) [34]. Representative sequences from each ASV were taxonomically annotated using the Naive Bayes classifier in QIIME2 against the SILVA database (version 138). To mitigate the influence of varying sequencing depths, all samples were rarefied to 20,000 sequences per sample for subsequent diversity and community analyses.

#### 2.2.4. Soil Physicochemical Analysis

Following the harvest and rhizosphere soil sampling, the air-dried soil samples from each plot were used to determine soil pH and exchangeable cation concentrations. Soil pH was measured potentiometrically in a 1:2.5 soil-to-water suspension. Exchangeable Ca^2+^ and Mg^2+^ were extracted with 1 M ammonium acetate (pH 7.0), and their concentrations were determined using Inductively Coupled Plasma Optical Emission Spectrometry (ICP-OES) [26].

### 2.3. Statistical Analysis

The Amplicon Sequence Variants (ASVs) obtained from the bioinformatic analysis represent the operational taxonomic units of the bacterial communities present in the rhizosphere soil. The relative abundance tables and taxonomic assignments derived from this process were the direct source of data for generating all figures and statistical analyses related to microbial community composition (e.g., bar charts, heatmaps, Venn diagrams) and diversity (e.g., beta-diversity indices, Principal Component Analysis (PCA), Principal Coordinates Analysis (PCoA), and Non-metric Multidimensional Scaling (NMDS)).

Based on the Bray–Curtis dissimilarity matrix, PCA, PCoA, and NMDS were performed to visualize differences in bacterial community structure among samples. Permutational multivariate analysis of variance (PERMANOVA) was performed using the adonis function with 999 permutations to test the significance of community structure differences between treatments. Linear Discriminant Analysis Effect Size (LEfSe) was employed to identify differentially abundant taxa (biomarkers) at the phylum to genus level across various treatments (LDA Score > 2.0, *p* < 0.05). All statistical analyses and visualizations were conducted primarily in the R programming environment (version 4.2.3), utilizing core packages including vegan (version 2.6-4), ggplot2 (version 3.4.4), and igraph (version 1.5.1) [35].

## 3. Results and Analysis

### 3.1. Characteristics of Soil Bacterial Community Composition

**Figure 1:**
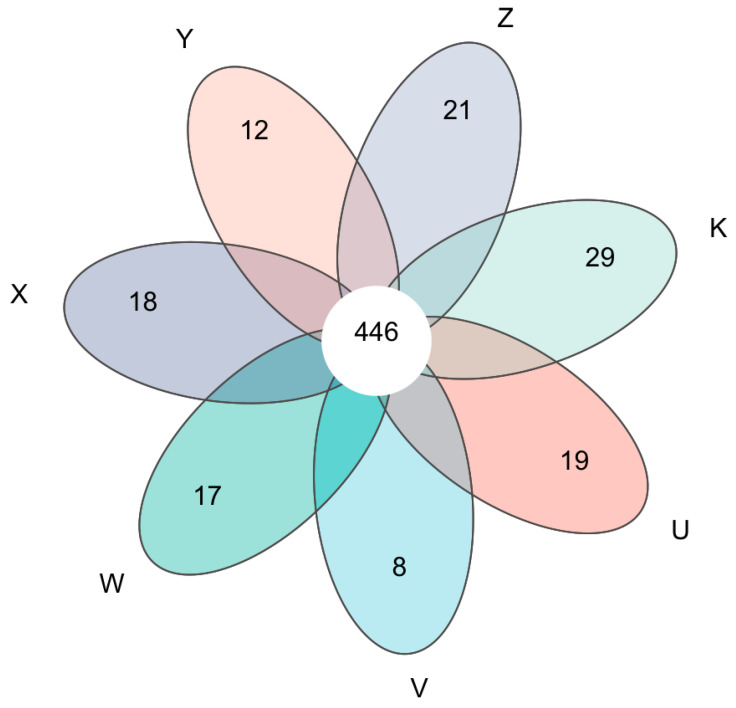
Venn diagram. Venn diagram showing shared and unique ASVs among treatments. Core ASVs (shared by all groups) and treatment-specific ASVs are indicated. U, V, W, X, Y, Z, and K represent different treatments. When ≥6 treatments are compared, a flower-petal diagram is shown: each petal displays the number of species unique to the corresponding treatment, and the center shows the number of species shared by all treatments. Due to the huge amount of data, which cannot be displayed one by one in the manuscript, we uploaded the core ASVs (shared by all treatments) and unique ASVs of each treatment, and their taxonomic affiliations to the system as an attachment (asv_taxon.xls) for easy reference.

The Venn diagram, by statistically analyzing the numbers of shared and unique ASVs among multiple treatments, intuitively revealed the similarity and specificity of bacterial communities under different Ca and Mg treatments. The analysis showed that the number of core ASVs shared by all treatments (U, V, W, X, Y, Z, K) was 446, primarily belonging to the genera *Bacillus, Pseudomonas, Streptomyces, and Sphingomonas*, representing a relatively high proportion of the total ASVs, indicating that Ca and Mg supplementation significantly altered the composition of the bacterial community in the rhizosphere soil. The number of unique ASVs per treatment was generally low, suggesting that supplementation treatments might promote the enrichment or disappearance of specific rare species through selective pressure. The number of pairwise or multiple overlapping ASVs between treatments (e.g., 9 shared ASVs between Y and Z) further indicated a gradient change in community structure. For instance, the high Ca & high Mg treatment (Z) shared more ASVs (21) with the high Mg treatment (X), suggesting that Mg addition might play a dominant role in driving community overlap; whereas the low Ca & low Mg treatment (Y) shared 12 ASVs with the low Ca treatment (U), reflecting a synergistic effect of low-level Ca (Figure 1). The patterns of ASV overlap between treatments (e.g., Y sharing more ASVs with U, Z sharing more with X) suggest that the degree of community similarity was influenced by the level and type of Ca and Mg supplementation. Overall, the Venn diagram emphasized that Ca and Mg supplementation not only reduced the core microbiome but also increased community beta-diversity, highlighting the key role of environmental filtering in shaping the microbial community in dryland soil.

**Figure 2:**
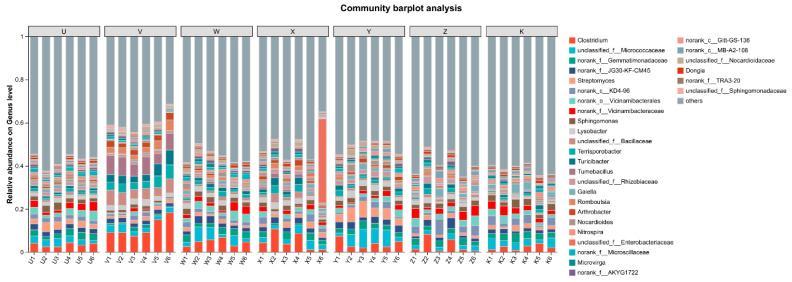
Community bar chart. Bar chart showing the relative abundance of dominant bacterial phyla and genera across treatments. U, V, W, X, Y, Z, K represent different treatments, each treatment is repeated 6 times (the same below). The *x*-axis shows sample names, and the *y*-axis shows the relative abundance of each taxon in the sample. Bars of different colors represent different taxa; bar length indicates the proportion of each taxon.

The community bar plot, based on taxonomic results (e.g., at the phylum or genus level), displayed differences in the relative abundance of dominant taxa among treatments. At the phylum level, *Bacillota* and *Actinobacteria* were dominant in all treatments, but their abundances were significantly affected by Ca and Mg supplementation (via Kruskal–Wallis test, *p* < 0.05). For example, the mean abundance of *Bacillota* was higher in the high Ca treatment (V, approx. 28.48%), while the abundance of *Actinobacteria* was relatively lower in the high Mg treatment (X, approx. 18.78%), suggesting that Ca addition might promote the enrichment of *Bacillota*, whereas Mg addition might inhibit *Actinobacteria*. The community composition in the control (K) was relatively balanced, while the combined treatments (e.g., Y and Z) showed intermediate abundances, indicating that Ca-Mg interactions might buffer the extreme effects of single elements. At the genus level, the abundances of *Clostridium* and *Streptomyces* varied significantly among treatments: the abundance of *Clostridium* was higher in the low Ca treatment (U), while the abundance of *Streptomyces* was lower in the high Mg treatment (X), potentially related to ion toxicity and nutrient competition (Figure 2). Principal Component Analysis (PCA) or Multivariate Analysis of Variance (MANOVA) could confirm that the overall differences in community composition between treatments were significant (*p* < 0.01). The bar plot results collectively indicate that Ca and Mg supplementation directly or indirectly regulated the composition and functional treatment distribution of the bacterial community in the rhizosphere of rainfed maize by altering soil chemical properties (e.g., Ca^2+^/Mg^2+^ ratio and pH).

**Figure 3:**
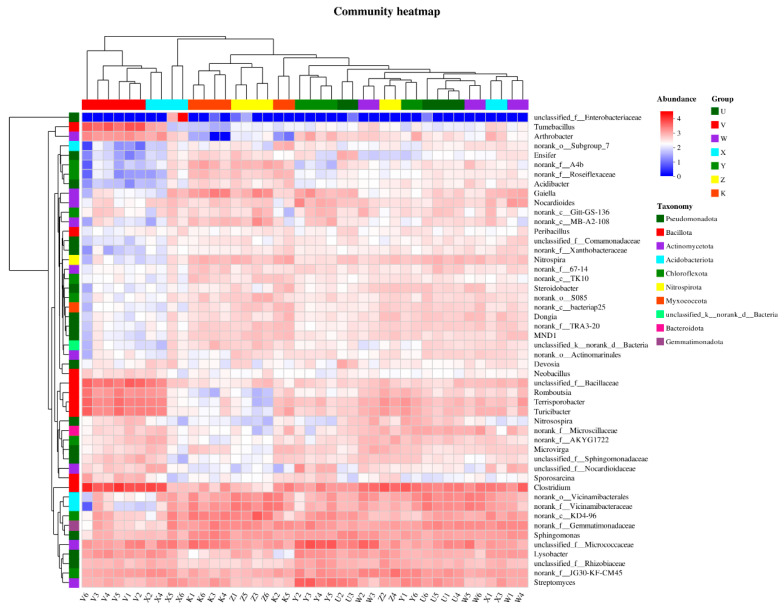
Community heatmap. Heatmap of top abundant bacterial genera, clustered by Bray–Curtis distance and Z-score normalized abundance. The heatmap was generated based on Z-score normalized relative abundances of the top 30 most abundant genera. Hierarchical clustering was performed using the Bray–Curtis distance and the UPGMA algorithm. The *x*-axis lists sample names, and the *y*-axis lists species names. A color gradient indicates the relative abundance of each species; the scale bar on the right shows the values corresponding to the gradient.

The heatmap visualized the distribution of top abundant taxa across different samples using a color gradient, combined with hierarchical clustering to show the bidirectional relationship between samples and taxa. The results showed that samples clustered into two main clusters based on treatment treatments: one cluster included the control (K) and low-level treatments (e.g., U and W), and the other included high-level treatments (e.g., V, X, Y, and Z), indicating that the level of Ca and Mg addition was the primary factor driving community differentiation. Regarding taxa, the heatmap revealed response patterns of specific genera. For instance, *Clostridium* showed high abundance in the high Ca treatment (V), while *Sphingomonas* was enriched in the low Mg treatment (W), suggesting that these taxa might serve as Ca- or Mg-sensitive biomarkers. Correlation analysis (e.g., Spearman’s rank correlation) could further verify significant positive or negative correlations between taxon abundance and Ca/Mg concentrations (*p* < 0.05). Furthermore, the color gradient in the heatmap showed that the high Ca & high Mg treatment (Z) had a unique species assemblage (e.g., high expression of *Turicibacter*), while the control (K) had a more uniform species distribution, reflecting the intensified selection pressure on community structure imposed by the supplementation treatments (Figure 3). Overall, the heatmap analysis emphasized that Ca and Mg supplementation led to heterogeneous distribution of taxa among samples by altering the micro-environment, providing a visual and statistical basis for understanding the ecological strategies of dryland soil microbes.

**Figure 4:**
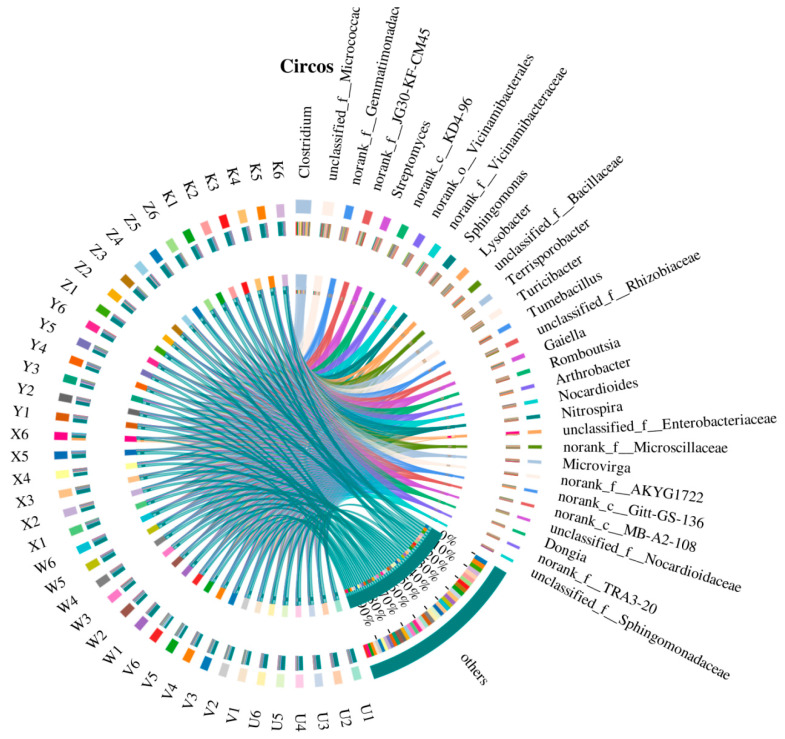
Circos diagram visualizing sample-taxon relationships in the rhizosphere bacterial community. The left semicircle (outer ring) represents samples, colored by treatment group (see legend). The inner ribbons on the left connect each sample to bacterial genera (right semicircle), with ribbon width proportional to the relative abundance of that genus in the sample. The right semicircle (outer ring) represents the dominant bacterial genera. The inner ribbons on the right show the distribution of each genus across different treatments (colored by treatment), with ribbon width indicating the proportion of the genus’s total abundance contributed by samples from that treatment. This visualization highlights treatment-specific enrichments (e.g., thick connections between High Ca treatment and *Clostridium*) and the sharing of taxa across treatments.

The Circos diagram displayed the correspondence between samples and dominant taxa in a circular layout, intuitively reflecting the proportion and distribution of community composition. In the diagram, the sample side (left half-circle) was colored by treatment, and the taxon side (right half-circle) represented the major dominant genera, with the width and color of the connecting ribbons indicating relative abundance. The analysis showed that the control (K) had widespread but weaker connections (thinner ribbons) to multiple taxa, indicating a more diverse community composition; whereas the high Ca (V) and high Mg (X) treatments had thick connections to specific taxa (e.g., *Clostridium* and *Streptomyces*), suggesting these treatments led to specialized enrichment of certain species. For example, the high Ca treatment (V) had a significant connection to Clostridium, potentially stemming from the promotion of cell membrane stability by calcium ions; while the low Mg treatment (W) had a strong connection to *Lysobacter*, possibly related to stress response under magnesium deficiency. Network analysis or abundance proportion tests could quantify the strength of sample-taxon associations, with results showing significant differences in distribution proportions among treatments (*p* < 0.01) (Figure 4). The Circos diagram also revealed Ca-Mg interactions: the species connection patterns in the combined treatments (Y and Z) were intermediate between the single treatments, suggesting that the added elements might regulate microbial resource allocation through competitive or synergistic mechanisms. In summary, the Circos diagram confirmed the reassembly of the rhizosphere bacterial community by Ca and Mg supplementation from a macro perspective, highlighting its potential regulatory function in dryland agricultural ecosystems.

### 3.2. Characteristics of Soil Bacterial Community Diversity

**Figure 5:**
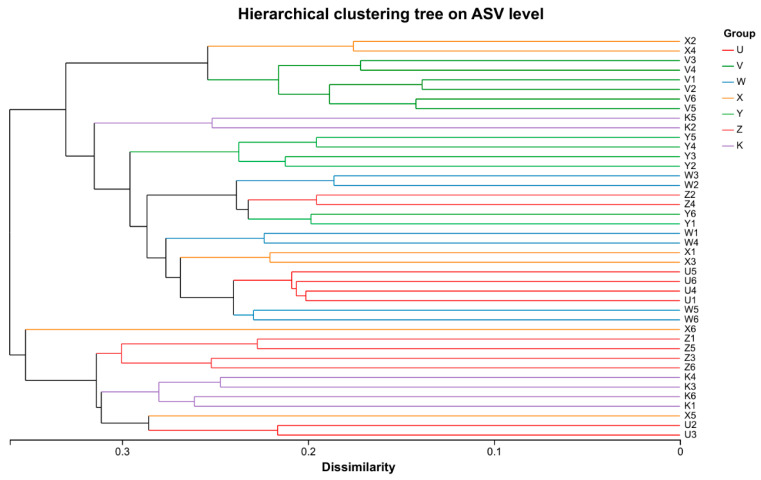
Hierarchical clustering tree of samples. Hierarchical clustering tree (UPGMA) of bacterial communities based on Bray–Curtis dissimilarity. Branch colors indicate treatment groups. The stacked bar chart (hidden by default) shows the relative abundance of dominant bacterial taxa. Branch length represents the distance between samples; distinct colors can indicate different treatments. When a stacked bar chart is displayed, the composition of dominant taxa in each sample is shown to the right of the clustering tree; by default, the stacked bar chart is hidden.

Hierarchical cluster analysis, based on a beta-diversity matrix (e.g., Bray–Curtis distance) and using the UPGMA algorithm, generated a dendrogram to visualize the similarity of community structures among samples. The results showed a clear clustering pattern according to treatment: control (K) samples aggregated into one major branch, while the Ca and Mg supplementation treatments (e.g., U, V, W, X, Y, Z) formed multiple sub-clusters. For instance, the low Ca (U) and high Ca (V) treatment samples clustered separately, indicating a significantly differentiating effect of Ca application level on community structure. Similarly, the low Mg (W) and high Mg (X) treatments also showed distinct clustering, suggesting that Mg addition likewise drives community variation. The combined treatments (Y and Z) were clustered interspersed with the single treatment treatments, reflecting the complexity of Ca-Mg interactions. Statistical testing (e.g., PERMANOVA) confirmed significant differences in the cluster structure between treatments (*p* < 0.05), supporting the role of Ca and Mg supplementation as key environmental factors altering the composition of the soil bacterial community (Figure 5). These results visually demonstrate that different Ca and Mg treatments led to higher similarity within treatments and lower similarity between treatments, highlighting the importance of Ca and Mg addition in shaping the microbial community in dryland soil.

**Figure 6:**
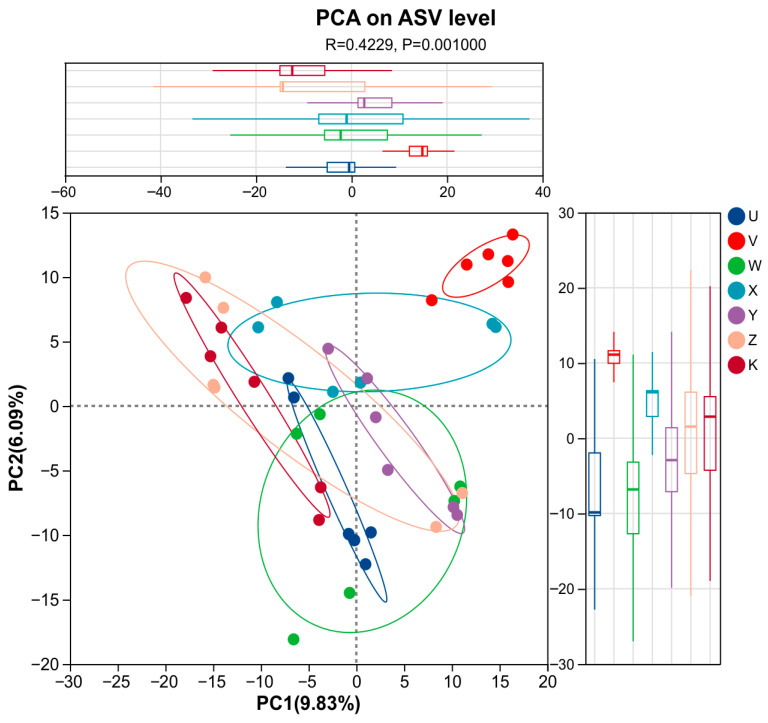
PCA plot. Principal Component Analysis (PCA) of bacterial communities based on Bray–Curtis distance. Percentages indicate the proportion of variance explained by each principal component. Sample points are colored by treatment. Different line colors represent the confidence intervals of the results in various treatments. The X- and Y-axes represent the two selected principal-component axes; the percentages indicate the proportion of variation in community composition explained by each component. Axis ticks show relative distances and carry no absolute meaning. Points of different colors or shapes denote samples from different treatments; the closer two points are, the more similar their species composition. If environmental factors are included, categorical variables are shown as points and continuous variables as vectors. The distance between a categorical-factor point and a sample point reflects the strength of that factor’s influence on the sample; the perpendicular distance from a sample point to a continuous-factor vector indicates the strength of that factor’s effect on the sample.

To assess differences in bacterial community structure (beta-diversity) among treatments, we employed Principal Coordinates Analysis (PCoA) and Non-metric Multidimensional Scaling (NMDS) as primary ordination methods, with Principal Component Analysis (PCA) also performed for comparison. PCA, based on the Bray–Curtis distance matrix, resulted in a relatively low cumulative variance explained by the first two principal components (PC1: 9.83%, PC2: 6.09%; cumulative 15.92%), indicating that a substantial portion of the community variation was not captured by this linear dimensionality reduction method. Nonetheless, an ANOSIM test confirmed significant differences between treatments (R = 0.4229, *p* = 0.001). The plot showed a partial separation, with control (K) samples clustered in the negative PC region and several amendment treatments (e.g., V, X) distributed in the positive region (Figure 6). Given the limited variance explained, PCA is presented here mainly for comparative purposes; primary interpretation of community structure separation relies on PCoA and NMDS.

**Figure 7:**
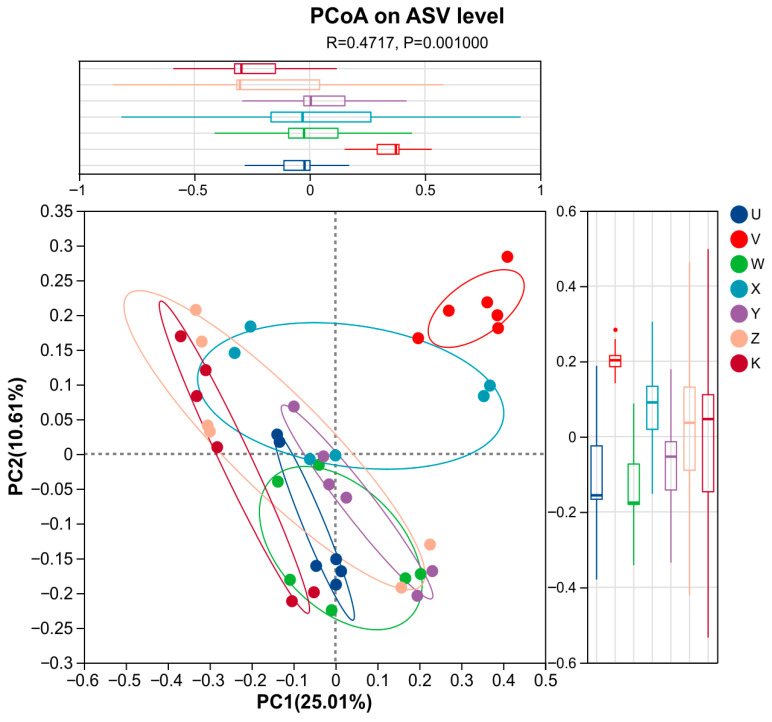
PCoA plot. Principal Coordinates Analysis (PCoA) based on Bray–Curtis distance. Percentages indicate the variance explained by each axis. The X- and Y-axes represent the two selected principal-coordinate axes; the percentages indicate the proportion of variation in community composition explained by each axis. Axis ticks show relative distances and carry no absolute meaning. Points of different colors or shapes denote samples from different treatments; the closer two points are, the more similar their species composition. Different line colors represent the confidence intervals of the results in various treatments.

PCoA analysis more effectively captured the community variation, with PC1 and PC2 explaining 25.01% and 10.61% of the variance, respectively (cumulative 35.62%). ANOSIM confirmed significant inter-treatment differences (R = 0.4717, *p* = 0.001). The PCoA plot revealed clear clustering of samples by treatment: the control (K) clustered on the left, while the high Ca & high Mg (Z) and high Mg (X) treatments were distributed on the right, and the low Ca & low Mg (Y) treatment was located towards the top. This suggests that the interactive effects of Ca and Mg addition led to community differentiation. For example, the high Ca (V) and high Mg (X) treatments were separated along the PC1 axis, hinting that Ca and Mg might influence community composition through different mechanisms, such as ion antagonism or synergy (Figure 7). The PCoA results further validated the findings from PCA and provided a clearer view of the community structure, highlighting the key role of Ca and Mg supplementation in shaping bacterial beta-diversity in the dryland soil.

**Figure 8:**
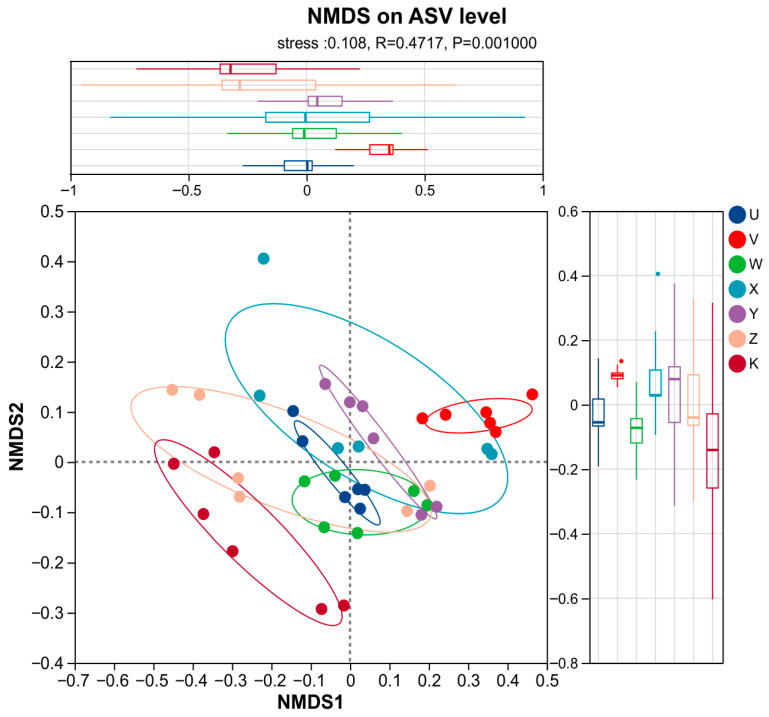
NMDS ordination. Non-metric Multidimensional Scaling (NMDS) ordination based on Bray–Curtis distance. Stress value = 0.108. Points of different colors or shapes represent samples from different treatments; the closer two points are, the more similar their species composition. Different line colors represent the confidence intervals of the results in various treatments. Axes display relative distances only and have no absolute meaning. Stress value: indicates the reliability of the NMDS solution. A stress < 0.2 is generally acceptable for a 2-D plot with interpretable patterns; stress < 0.1 indicates a good fit; stress < 0.05 represents an excellent representation. The stress value shown is rounded to three decimal places.

NMDS analysis employed a non-parametric multidimensional scaling method. The stress value was 0.108 (less than 0.2), indicating a good fit for the dimensionality reduction and reliable results. ANOSIM tests revealed significant differences between treatments (R = 0.4717, *p* = 0.001), which is consistent with the PCoA results. NMDS analysis (stress = 0.108) corroborated the patterns observed in PCoA, showing clear treatment-based clustering: the control treatment (K) was concentrated in a compact area, while Ca and Mg supplementation treatments, such as high Ca (V) and high Mg (X) were scattered peripherally, forming independent clusters. The low-level treatments (U and W) and the combined treatments (Y and Z) showed overlapping distributions, reflecting a gradient in community response (Figure 8). NMDS, utilizing distance matrices (e.g., *UniFrac*), emphasized the differences in community structure between treatments. For instance, the high Ca treatment might enrich for specific tolerant bacterial treatments, while the low Mg treatment might promote opportunistic species. Overall, the NMDS analysis reinforced the beta-diversity results, confirming that Ca and Mg supplementation significantly altered the structure of the bacterial community in the rhizosphere soil of rainfed maize, and that these changes were closely related to the application level and type of supplementation.

### 3.3. Characteristics of Soil Bacterial Community Species Differentiation

**Figure 9:**
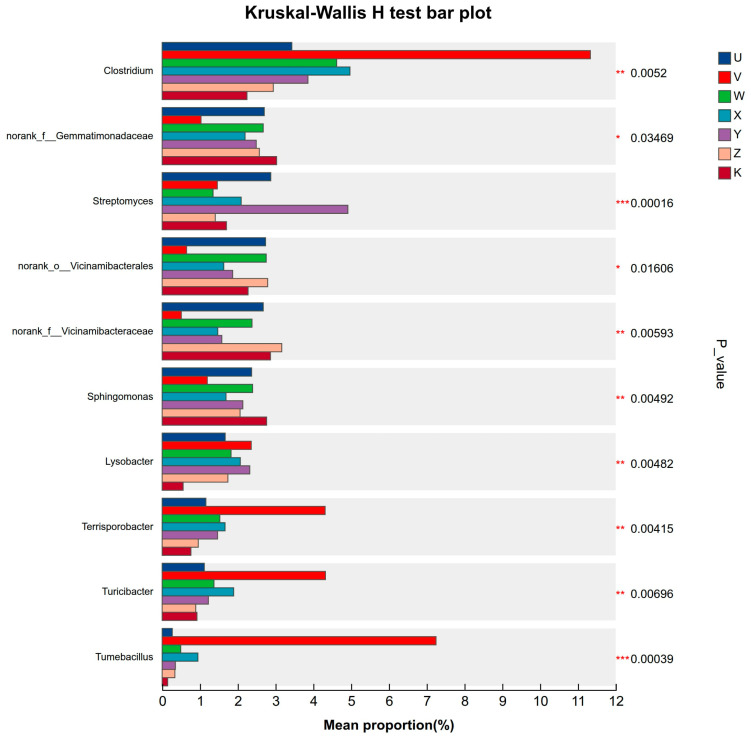
Multi-species differential-abundance test. Box plots showing significant differences in relative abundance of key bacterial genera across treatments (Kruskal–Wallis test, *p* < 0.05). The *y*-axis lists taxon names at the selected taxonomic level; the *x*-axis shows the mean relative abundance of each taxon across treatments. Bars of different colors represent different treatments. *p*-values are displayed on the far right: * 0.01 < *p* ≤ 0.05, ** 0.001 < *p* ≤ 0.01, and *** *p* ≤ 0.001.

Multi-treatment comparison analysis employed the non-parametric Kruskal–Wallis H test to conduct hypothesis testing on the relative abundance of specific taxa across multiple sample treatments, assessing the significance of differences. Results from bar plots and box plots collectively showed that Ca and Mg supplementation treatments significantly altered the abundance of several key taxa (*p*-values ranging from 0.0001532 to 0.02469). For instance, the genus *Clostridium* exhibited the highest mean relative abundance in the high Ca treatment (V), with highly significant differences (*p* = 0.0002) compared to the control (K) and most other treatments. This indicates that a high-calcium environment might be particularly favorable for the proliferation of these bacteria, which are often associated with anaerobic metabolism. The abundance of *Streptomyces* was significantly reduced (*p* = 0.0001562) in the low Ca & low Mg treatment (Y), suggesting that concurrent deficiency of Ca and Mg might synergistically inhibit the growth of these actinobacteria, known for antibiotic production. Furthermore, genera such as *Turicibacter* and *Terrisporobacter* also showed significant differences (*p* < 0.01) in specific treatments (e.g., high Ca & high Mg). Post hoc tests (e.g., Dunn’s test) could further clarify the differences in pairwise comparisons between treatments (Figure 9). These results clearly demonstrate that Ca and Mg supplementation did not uniformly affect all microorganisms but exerted specific selective pressures on certain functional treatments, likely mediated through changes in soil pH, ionic strength, or nutrient availability. These significantly different taxa are potential biomarkers for indicating the management status of calcium and magnesium nutrients in the soil.

**Figure 10:**
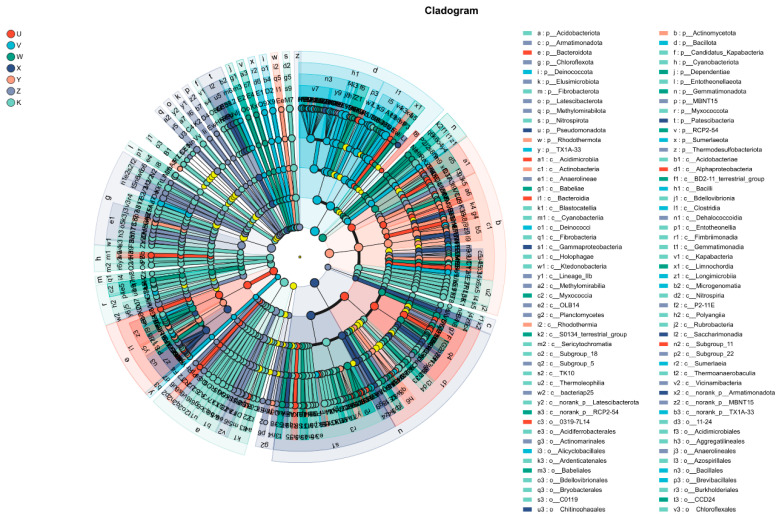
LEfSe multi-level cladogram. Colored nodes indicate microbial taxa that are significantly enriched in the corresponding treatment and contribute strongly to inter-treatment differentiation; light-yellow nodes represent taxa that show no significant enrichment in any treatment or do not significantly influence inter-treatment differences.

LEfSe (LDA Effect Size) analysis identified biomarkers that were significantly enriched in different treatments and contributed most to inter-treatment differences, through multi-level statistical testing from phylum to genus (or even species). A hierarchical cladogram visually displayed the distribution of these differential taxa in a phylogenetic format. The results showed that different Ca and Mg treatments selectively enriched specific taxonomic units. For example, in the high Ca treatment (V), the class Clostridia under the phylum *Firmicutes* and its affiliated families and genera were significantly marked. This corroborates the enrichment of *Clostridium* found in the multi-treatment comparison analysis, indicating that the high-calcium environment created a favorable ecological niche for these Gram-positive, potentially spore-forming bacteria. Conversely, in the high Mg treatment (X), the class *Gammaproteobacteria* under the phylum *Proteobacteria* and genera like *Lysobacter* were identified as key differential species. The control (K) was enriched with some species from the phylum *Actinobacteria* (Figure 10). Branches of different colors in the cladogram clearly marked the enrichment of these species in different treatments, while yellow nodes indicated taxa that did not show significant inter-treatment differences at certain taxonomic levels. This analysis revealed that the impact of Ca and Mg supplementation permeated various taxonomic levels of microbial treatments, emphasizing the importance of multi-level analysis when assessing the microbial effects of fertilization strategies.

**Figure 11:**
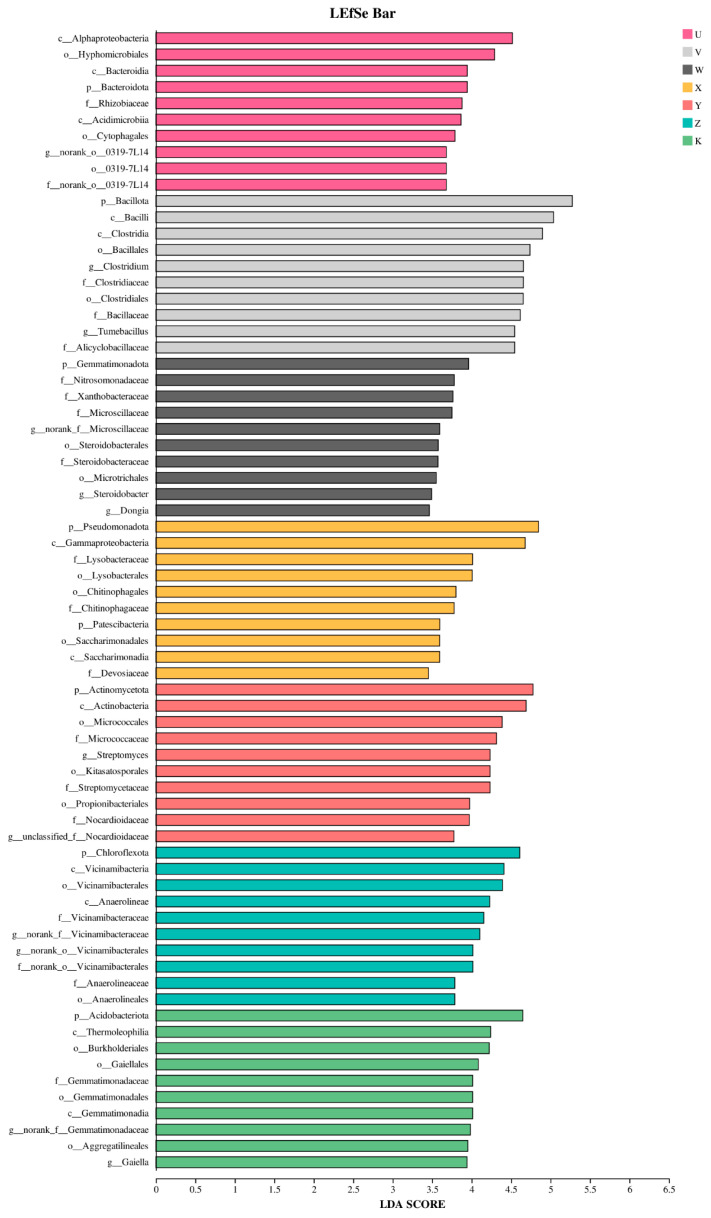
LDA effect size (LEfSe) bar plot. The *x*-axis shows the linear discriminant analysis (LDA) score (proportional to the logarithmic abundance of each taxon), the *y*-axis lists taxon names at the selected taxonomic rank, and bars are color-coded by treatment.

The LDA bar chart quantitatively presents the results of the LEfSe analysis, showing the statistically significant biomarkers and their LDA scores (Linear Discriminant Analysis Score). This score measures the effect size of each taxon in differentiating the treatments (typically, an LDA score > 2.0 or 3.0 is set as the significance threshold). The bar chart shows that different treatments possessed unique signature microbial communities. For instance, in the high Ca treatment (V), the genus Clostridium and related family and class-level taxa obtained high LDA scores (generally > 4.0), confirming them as the most strongly responsive indicators to Ca addition. In the high Mg treatment (X), the family *Lysobacteraceae* and the genus *Lysobacter* exhibited relatively high LDA scores (~3.5), indicating their enrichment under Mg addition contributed significantly to community differentiation (Figure 11). Notably, in the high Ca & high Mg combined treatment (Z), genera like *Turicibacter* also showed relatively high LDA values, suggesting that unique interactive effects might select for specific microbial combinations when Ca and Mg coexist. These LDA scores form a “microbial fingerprint” that can clearly distinguish the different Ca and Mg supplementation treatments. By comparing the magnitude of the LDA scores, these biomarkers can be prioritized; those with higher scores have greater potential as characteristic indicator species for their respective treatments and warrant focused attention in subsequent predictive modeling or mechanistic studies.

### 3.4. Soil Physicochemical Properties in Response to Ca and Mg Addition

The application of Ca and Mg fertilizers significantly altered the soil chemical properties in the maize rhizosphere (Table 1). As expected, the high Ca treatment (V) and the high Ca & high Mg treatment (Z) led to a significant increase in exchangeable Ca^2+^ compared to the control (K) and low Mg treatments (W, X). Similarly, the high Mg treatment (X) and the high Ca & high Mg treatment (Z) significantly increased the exchangeable Mg^2+^ concentration. A slight but non-significant increase in soil pH was observed in Ca-amended treatments, consistent with the basifying effect of calcium ions. These confirmed chemical changes provide a direct environmental context for the observed shifts in the bacterial community structure, firmly linking the fertilization treatments to the modified soil microenvironment.

The observed increments in soil pH following Ca addition, albeit statistically non-significant in our study (likely due to the high initial buffering capacity of the calcareous soil), were consistent with the theoretical basifying effect of Ca^2+^. In high-pH environments, even marginal shifts would influence ion speciation and bioavailability. More critically, the significant and substantial increases in exchangeable Ca^2+^ and Mg^2+^ concentrations (Table 1) represented a direct and major alteration of the ionic milieu. This change in cation loading and the resultant shifts in Ca^2+^/Mg^2+^ ratios were likely more ecologically relevant drivers of microbial community restructuring in this system than the absolute pH change, affecting membrane stability, enzyme activity, and ionic balance for microorganisms.

## 4. Discussion

This study, through the integrated application of high-throughput sequencing and multivariate statistical analyses, systematically revealed the significant effects of different calcium (Ca) and magnesium (Mg) supplementation treatments on the structure, diversity, and specific taxon abundance of the bacterial community in the rhizosphere soil of rainfed maize. Our findings not only confirm the sensitivity of soil microbial communities to changes in environmental factors but also deepen our understanding of the roles played by Ca and Mg ions in the rhizosphere micro-ecosystem [36].

### 4.1. Ca and Mg Supplementation as Key Regulators Reshape Rhizosphere Bacterial Community Structure

This study found that different Ca and Mg supplementation treatments significantly altered the beta-diversity of the bacterial community in the rhizosphere soil of rainfed maize, while the response of alpha-diversity was more complex [37]. PCA, PCoA, and NMDS analyses consistently demonstrated significant separation between treatments and the control in the ordination space (*p* < 0.01), clearly revealing the profound impact of Ca and Mg input on the assembly of the rhizosphere microbial community [38]. This result strongly supports the environmental filtering theory. The introduction of calcium (Ca^2+^) and magnesium (Mg^2+^) ions likely established a potent abiotic selective barrier by altering the pH, ionic strength, aggregate structure, and organic matter complexation status within the rhizosphere microzone [39]. Under this barrier, taxa with corresponding physiological adaptations (e.g., those tolerant of high osmotic pressure or preferring specific pH ranges) became enriched, while sensitive taxa were suppressed, ultimately leading to directional succession in the overall community structure. Regarding alpha-diversity, the slightly higher ASV numbers in the low-level Ca and Mg treatments compared to the control might align with the “Intermediate Disturbance Hypothesis” suggesting that moderate nutrient disturbance creates more diversified microhabitats [40,41]. In contrast, the lack of a significant increase in diversity under high-level or combined treatments implies that overly strong selective pressure or complex ion antagonism might limit species coexistence. This is consistent with numerous studies on the effects of fertilization on microbial diversity, indicating that the effect of nutrients is clearly level-dependent [42].

### 4.2. Specific Responses of Key Taxa Reveal Potential Microbial Ecological Functions

Using LEfSe analysis and multi-treatment comparisons, this study successfully identified key biomarkers responsive to different Ca and Mg treatments, which constitute a “microbial fingerprint” for each treatment. For instance, the high Ca treatment significantly enriched the genus *Clostridium* sensu lato (phylum Firmicutes). It is crucial to note the considerable functional heterogeneity within this genus. While some *Clostridium* species are pathogenic or strict anaerobes, many are facultative anaerobes and key participants in soil organic matter decomposition, fermentation, and nitrogen fixation [43]. Their enrichment might suggest an alteration in micro-niche oxygen dynamics or substrate availability influenced by Ca-mediated soil aggregation. However, without metagenomic or transcriptomic data, we caution against assigning a specific functional role and instead highlight this taxon as a calcium-responsive biomarker [44].

Conversely, the high Mg treatment significantly enriched the genus *Lysobacter* (Gammaproteobacteria). Members of this genus are frequently reported in the literature as prolific producers of lytic enzymes and antibiotics, contributing to pathogen suppression and chitin degradation [45]. Based on these well-documented traits, its enrichment could indicate a potential enhancement of the rhizosphere’s disease-suppressive capacity or organic matter turnover linked to Mg availability. Future research targeting functional genes is needed to confirm these hypothesized linkages.

We acknowledge that the enrichment of specific bacterial taxa may not solely result from direct ionic effects on microbial physiology (environmental filtering). An alternative or complementary pathway involves plant-mediated indirect selection. Alterations in Ca^2+^ and Mg^2+^ availability can influence plant physiology, root exudation patterns, and the quantity/quality of organic carbon released into the rhizosphere [26]. These modified exudates then act as primary substrates and signaling molecules, selectively stimulating or inhibiting specific microbial populations. Therefore, the observed biomarker taxa likely reflect the integrated outcome of both direct ionic effects on microbes and indirect effects mediated through changes in plant root ecology.

### 4.3. Complexity of Ca-Mg Interactions Highlights Limitations of Single-Nutrient Studies

A noteworthy finding deserving further exploration is that the combined effects of Ca and Mg were not simply additive to their individual effects. For example, in terms of community structure, both the low Ca & low Mg and high Ca & high Mg treatments exhibited unique clustering patterns, rather than falling entirely within clusters dominated solely by Ca or Mg [46]. The Venn diagram also showed unique patterns of shared and unique ASVs for the combined treatments. This indicates that complex interactions likely exist between Ca^2+^ and Mg^2+^ in the rhizosphere microzone, potentially including competition for plant root uptake sites, synergistic promotion, or antagonistic effects on soil colloid stability [47]. The overwhelming focus of current research on the effects of single elements (e.g., applying only Ca or only Mg) largely overlooks such nutrient interactions, which might be one reason for inconsistencies among different studies. This finding underscores that in future practices aimed at managing soil microbes through nutrient management, a shift in mindset from single-nutrient management to multi-nutrient collaboration management is necessary, fully considering elemental stoichiometric balance and biological interactions to more accurately predict and manage soil microbial community responses [48].

The results of the soil chemical analysis confirm the successful establishment of the intended nutrient gradients. The significant increases in exchangeable Ca^2+^ and Mg^2+^ in their respective treatments provide a clear mechanistic link between fertilization and the observed microbial shifts. The changes in community structure, as revealed by beta-diversity analysis, are therefore a direct consequence of this altered soil chemical environment, reinforcing the environmental filtering hypothesis [49].

From a practical agronomic perspective, our identification of treatment-specific microbial biomarkers (e.g., *Clostridium* for high Ca, *Lysobacter* for high Mg) provides a potential ‘microbial fingerprint’ for diagnosing soil Ca/Mg status or forecasting community shifts. While it is premature to define precise agronomic fertilization thresholds for deliberate microbiome engineering based on a single study, our findings establish a proof-of-concept. They indicate that Ca and Mg management could be leveraged, alongside other practices, to nudge rhizosphere communities towards desired functional states—for instance, potentially promoting taxa associated with disease suppression (*Lysobacter*) via Mg supplementation [50]. The non-additive interactions further underscore that such management would require a balanced, multi-nutrient approach rather than single-element adjustments.

This study provides a robust snapshot of community assembly at the maize maturity stage. However, as a single-time-point observation, it cannot capture the temporal dynamics and successional trajectories of the microbial community following Ca and Mg supplementation. Rhizosphere communities are known to shift with plant phenology. Future studies employing longitudinal sampling across key growth stages are essential to determine if the observed treatment effects are consistent, transient, or amplified over time. Furthermore, to move beyond correlation and substantiate the ecological functions inferred for biomarker taxa (e.g., *Clostridium*, *Lysobacter*), integrated multi-omics approaches are recommended. Shotgun metagenomics would reveal the presence of functional genes related to nutrient cycling and pathogen antagonism [51]. Metatranscriptomics or metabolomics, coupled with root exudate profiling, could elucidate active microbial pathways and their chemical dialog with the host plant, directly linking specific Ca/Mg-induced changes in the root environment to microbial functional responses.

Additionally, this study utilized a single, high-performing maize hybrid (‘Haodan 168’) to control for plant genetic variation. While this strengthens the internal validity of our findings regarding Ca/Mg effects, it necessarily limits exploration of plant genotype–microbiome interactions. Different plant genotypes can recruit distinct rhizosphere communities through varied exudate profiles. Future research should examine whether the Ca/Mg-responsive microbial patterns identified here are conserved across maize varieties or are genotype-specific. This knowledge is critical for developing broadly applicable or genotype-tailored nutrient management strategies aimed at microbiome optimization.

## 5. Conclusions

This study systematically elucidated the profound impacts of calcium (Ca) and magnesium (Mg) supplementation on the bacterial community in the rhizosphere soil of rainfed maize. The main conclusions were as follows:

(1) Calcium and magnesium supplementation served as key environmental drivers of rhizosphere bacterial community differentiation. This study confirmed that inputs of Ca and Mg at different levels and combinations created potent environmental filtering pressures by altering the rhizosphere microenvironment, leading to significant and specific restructuring of the bacterial community structure. The community response was primarily reflected in beta-diversity.
(2) Different treatments enriched specific key bacterial taxa, which can serve as potential biomarkers. Through multi-treatment comparisons and LEfSe analysis, we identified treatment-specific “microbial fingerprints,” such as the enrichment of Clostridium under high Ca treatment and *Lysobacter* under high Mg treatment. The enrichment of these key taxa indicates potential shifts in specific ecological functions guided by Ca and Mg supplementation, such as enhanced potential for anaerobic organic matter decomposition or disease suppression. This provides concrete targets for fostering beneficial rhizosphere microbiota through targeted nutrient management.
(3) Complex interactions existed between calcium and magnesium, and their combined effects were not simply additive. The combined Ca-Mg treatments resulted in unique community compositions that were not merely the sum of their individual effects. This finding underscores the necessity to shift from an isolated perspective to an integrated one in future soil nutrient management and microbial ecology research, fully accounting for nutrient synergism and antagonism.

From a microbial ecology perspective, this study confirms that appropriate calcium and magnesium management is an effective strategy for regulating and improving soil health in dryland farming systems.

## Figures and Tables

**Table 1:** Post-harvest soil pH and exchangeable Ca^2+^ and Mg^2+^ concentrations in the rhizosphere of maize under different fertilization treatments. (Values are mean ± standard error; n = 4. Different letters within a column indicate significant differences at *p* < 0.05 according to Duncan’s test.).

Treatment	pH (H_2_O)	Exchangeable Ca^2+^ (cmol_(+)_∙kg^−1^)	Exchangeable Mg^2+^ (cmol_(+)_∙kg^−1^)
K (Control)	8.52 ± 0.04 ^ab^	4.85 ± 0.21 ^c^	1.25 ± 0.08 ^c^
U (Low Ca)	8.55 ± 0.03 ^ab^	5.92 ± 0.18 ^b^	1.28 ± 0.09 ^c^
V (High Ca)	8.59 ± 0.05 ^a^	7.41 ± 0.32 ^a^	1.31 ± 0.11 ^c^
W (Low Mg)	8.50 ± 0.04 ^b^	4.91 ± 0.25 ^c^	1.87 ± 0.14 ^b^
X (High Mg)	8.51 ± 0.03 ^ab^	4.99 ± 0.19 ^c^	2.65 ± 0.19 ^a^
Y (Low Ca & Low Mg)	8.53 ± 0.04 ^ab^	5.88 ± 0.22 ^b^	1.90 ± 0.12 ^b^
Z (High Ca & High Mg)	8.57 ± 0.04 ^a^	7.35 ± 0.28 ^a^	2.58 ± 0.17 ^a^

## Data Availability

The original contributions presented in this study are included in the article. Further inquiries can be directed to the corresponding author.

## Abbreviations

The following abbreviations are used in this manuscript:

U	Low Ca
V	High Ca
W	Low Mg
X	High Mg
Y	Low Ca & Low Mg
Z	High Ca & High Mg
K	No Ca and No Mg
